# Penicillin allergy evaluation in hospitalized patients with hematologic malignancy

**DOI:** 10.1017/ash.2023.144

**Published:** 2023-05-16

**Authors:** Vima M. Patel, Brian Chu, Keith W. Hamilton, Cassandra Bellamy, Christina Harker, Joshua S. Bryer, Bridget Shields, Rebecca L. Hirsh, Olajumoke O. Fadugba, Robert G. Micheletti

**Affiliations:** 1 Section of Allergy and Immunology, Division of Pulmonary Allergy and Critical Care, Perelman School of Medicine, University of Pennsylvania, Philadelphia, Pennsylvania; 2 Perelman School of Medicine, University of Pennsylvania, Philadelphia, Pennsylvania; 3 Division of Infectious Diseases, Department of Medicine, Perelman School of Medicine, University of Pennsylvania, Philadelphia, Pennsylvania; 4 Department of Pharmacy, Hospital of the University of Pennsylvania, Philadelphia, Pennsylvania; 5 Division of Hematology/Oncology, Department of Medicine, Perelman School of Medicine, University of Pennsylvania, Philadelphia, Pennsylvania; 6 Department of Dermatology, Perelman School of Medicine, University of Pennsylvania, Philadelphia, Pennsylvania; 7 Department of Dermatology, University of Wisconsin, Madison, Wisconsin; 8 Department of Medicine, Perelman School of Medicine, University of Pennsylvania, Philadelphia, Pennsylvania

## Abstract

A penicillin allergy testing service (PATS) assessed penicillin allergy in patients with hematologic malignancies; 17 patients who met criteria had negative skin testing. Patients who underwent penicillin challenge passed and were delabeled. Of delabeled patients, 87% received and tolerated β-lactams during follow-up. Providers found the PATS valuable.

Penicillin-class antibiotics are the most common cause of drug allergy, reported in up to 15% of the inpatient population.^1,2^ Those with hematologic malignancies are at high risk of infection, and β-lactam antibiotics are frequently recommended as empiric therapy.^3,4^ Inpatients with hematologic malignancy and β-lactam allergy have increased antibiotic use and mortality risk, higher infection and readmission rates, and longer hospital length of stay than those without β-lactam allergy, likely due to use of nonpreferred antibiotics.^5,6^

Most patients with penicillin-class allergy label are not truly penicillin allergic, and true penicillin allergy can wane with extended avoidance. At least 90% of previously allergic patients have negative penicillin skin testing (PST) and tolerate penicillin-class antibiotics.^2^ Penicillin allergy testing is safe and effective in inpatient and outpatient settings and is encouraged by professional societies and health agencies.^7,8^ We evaluated the feasibility of implementing penicillin allergy testing guidelines in hospitalized patients with hematologic malignancies and reported penicillin-class allergy. We aimed to improve access to β-lactam antibiotics. Additionally, we surveyed hematology-oncology providers on the acceptability of a penicillin allergy testing service (PATS).

## Methods

The PATS, consisting of an allergist and nurse practitioner, was formed to evaluate penicillin allergy in hospitalized patients with hematologic malignancy. Patients on the hematology service were screened for penicillin or amoxicillin allergy labels using the electronic health record (EHR). Exclusion criteria included reaction within the last 10 years, history of severe cutaneous adverse reaction (eg, Stevens-Johnson syndrome), history of non-IgE mediated reaction, hemodynamic instability, pregnancy, concomitant antihistamine use, and severe acute respiratory coronavirus virus 2 (SARS-CoV-2) positivity. Once identified, the PATS reviewed the study aims with the patient’s primary hospital team and obtained permission to proceed. The PATS surveyed patients to characterize their allergy history (Supplementary Fig. 1). Patients with an IgE-mediated or unknown reaction who met criteria for testing underwent standardized PST by an allergist as soon as it could be coordinated. Those with negative PST underwent incremental aminopenicillin challenge with nursing assistance. If the skin test was negative and the challenge was tolerated, the results of the evaluation were documented and the allergy label was removed from the EHR after discussion with the care team and patient. The primary outcome was the proportion of patients whose allergy label was removed following PATS evaluation. A secondary outcome was subsequent receipt of β-lactam antibiotics. Hematology-oncology providers, nursing staff, and pharmacists were surveyed to evaluate the acceptability of the PATS.

## Results

**Fig. 1.**
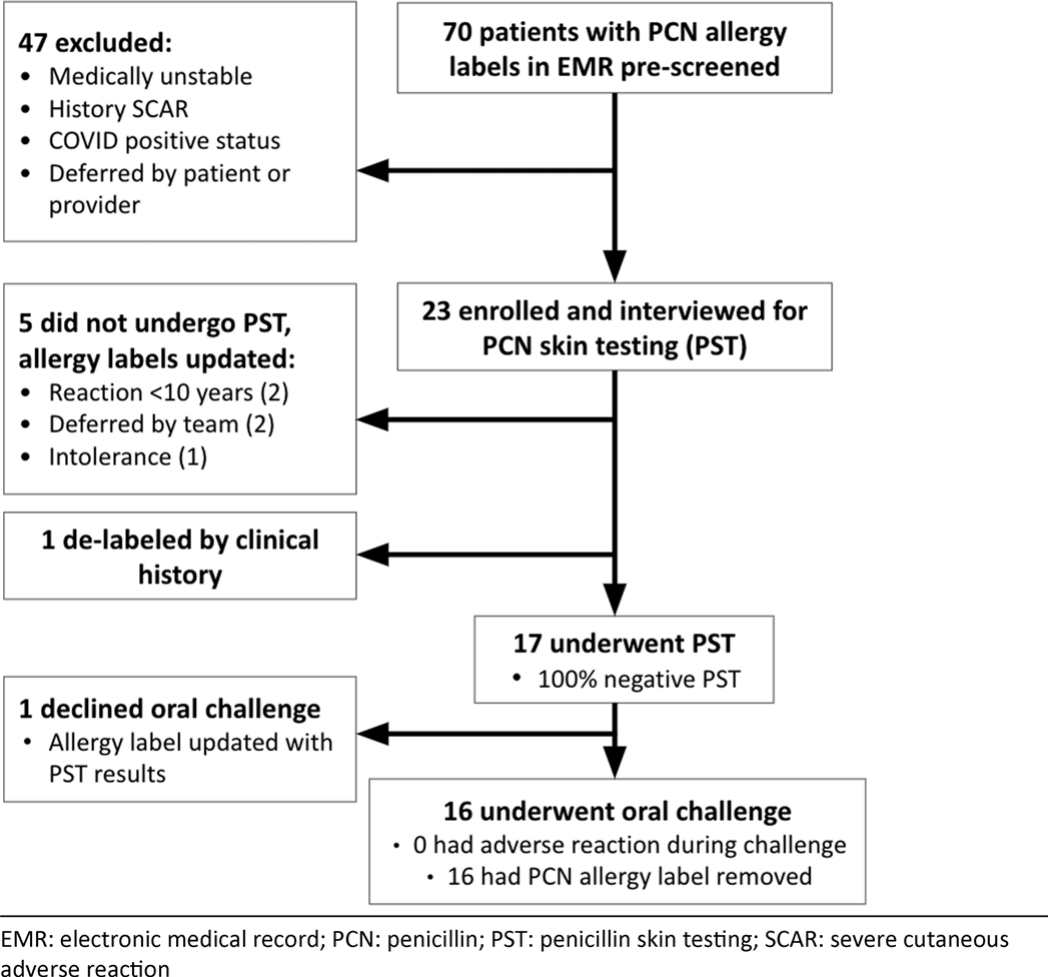
Flowchart of screened and enrolled patients.

Between November 2020 and April 2021, 70 hospitalized patients with hematologic malignancy and penicillin or amoxicillin allergy were identified. Among them, 47 patients were excluded due to hemodynamic instability, concomitant antihistamine use, SARS-CoV-2 positivity, or deferment by the primary hospital team or patient (Table 1). Of the 23 patients who consented to participate, the median age was 61 years, and 20 patients (87%) were actively receiving chemotherapy for a range of hematologic malignancies (Fig. 1). Penicillin and amoxicillin allergy were reported in 11 patients (48%) and 3 patients (13%), respectively. The precise penicillin-class antibiotic was uncertain in 9 patients (39%). Patients’ description of penicillin allergy varied, including rash, anaphylaxis, chest pain, and fatigue (Table 1).

**Table 1.** Patient Demographics

Patient Demographics	Total (N = 23), No. (%)^a^
Age, median y (range)	61 (26–72)
**Sex**	
Male	12 (52.2)
Female	11 (47.8)
**Race**	
White	17 (73.9)
Black	5 (21.7)
Unknown	1 (4.4)
**Medical History**	
**Medications (n = 23)**	
Corticosteroids	15 (65.2)
Beta blocker	2 (8.7)
Chemotherapy	20 (87.0)
**Indications for chemotherapy (n = 20)**	
Multiple myeloma	7 (35)
AML	3 (15)
DLBCL	3 (15)
ALL	3 (15)
T-cell lymphoma	2 (10)
MDS	1 (5)
Primary amyloidosis	1 (5)
**Patient description of penicillin allergy label (n = 23)**	
Rash	13
Hives	7
Facial swelling	1
Shortness of breath	1
Unknown	2
Anaphylaxis	1
Chest Pain	1
Fatigue	1

Note. AML, acute myeloid lymphoma; DLBCL, diffuse large B-cell lymphoma; ALL, acute lymphoid leukemia; MDS, myelodysplastic syndrome.

a Units unless otherwise specified.

After obtaining a relevant history, 17 (74%) of the 23 enrolled patients met criteria for PST. The remaining 6 patients either did not meet criteria for testing (n = 5) or had allergy labeling removed based on clinical history alone (n = 1). Of the 17 patients tested, 100% had negative PST, and 16 underwent and passed an incremental aminopenicillin oral challenge. One patient declined the challenge despite negative PST. After tolerating the challenge, all 16 patients agreed to penicillin allergy delabeling. During a 3-month follow-up period, 14 of 17 tested and delabeled patients received β-lactam antibiotics. Penicillins and cephalosporins were administered to 3 and 11 patients, respectively, for indications including neutropenic fever, sepsis, cellulitis, joint infection, dental prophylaxis, urinary tract infection, and pneumonia. These agents were all considered firstline choices for their respective indications. Cefepime was tolerated by the 1 patient who had negative PST but declined the challenge. None experienced an allergic reaction to a β-lactam antibiotic.

The feasibility and acceptability of the PATS was evaluated via a follow-up survey administered to hematology-oncology physicians, advanced practice providers, nurses, and pharmacists (Supplementary Fig. 2). Of 17 providers, 12 (71%) rated their understanding of the purpose of the study as “very good,” and all described communication between the PATS and the clinical team as “very clear” (10 of 17, 59%) or “moderately clear” (7 of 17, 41%). Providers characterized the clinical team as “completely” receptive to patients undergoing penicillin testing (10 of 17, 59%) or “moderately” receptive to patients undergoing penicillin testing (7 of 17, 41%) and “completely” receptive to removing patient allergy labels based on negative testing (15 of 17, 88%) or “moderately” (2 of 17, 12%) receptive to removing patient allergy labels based on negative testing.

Of 22 providers, 9 (41%) felt that participation in penicillin testing would interfere with their patient’s wellness; specifically, 2 (9%) of 22 providers believed that their patient was too sick for testing, and 7 (32%) of 22 providers were concerned that testing might conflict with other treatments. In 3 (23%) of 13 survey responses, providers reported that some patients were initially reluctant to remove allergy labeling due to fear they might still be allergic to penicillin despite negative testing. Also, 1 patient (8%) did not trust the process used to determine penicillin allergy status, and 1 patient (8%) preferred to decide after discussion with their primary physician or oncologist. Of 18 providers, 17 (94%) felt that the PATS was very valuable for patients, and 15 (83%) felt that a dedicated team routinely performing penicillin allergy evaluation for hospitalized patients would be very valuable.

## Discussion

Penicillin allergy delabeling programs have been implemented in outpatient and inpatient settings, demonstrating safety and improved outcomes.^7,9^ In these programs, clinical pharmacists, hospitalists, allergists, or infectious diseases providers screen, evaluate, and test for penicillin allergy. Because penicillin allergy is associated with poorer outcomes in those with hematologic malignancies,^5^ such patients are particularly likely to benefit from routine verification of penicillin allergy and delabeling, when appropriate.

Despite potential benefits, studies of PST have often excluded this patient population. The safety and efficacy of routine penicillin allergy evaluation have been demonstrated in patients with leukemia and genitourinary malignancies.^10^ Our study focused on patients with hematologic malignancy, and we gathered data on acceptability of the intervention by hematology-oncology providers. Overall, 74% of patients had their penicillin allergy label removed and 82% of these received and tolerated β-lactam antibiotics following delabeling as part of their routine care. The high rate of β-lactam use in the 3 months following the PATS intervention demonstrates the frequent need of such antibiotics in patients with hematologic malignancy, highlighting the importance of the intervention.^3,4^

Implementation of the PATS was received positively by providers, who expressed a willingness to make clinical decisions based on the results of penicillin allergy testing. Clear communication between the PATS and the primary hospital team, as well as a high level of understanding of the value of penicillin allergy evaluation, were essential to the success of the program. The fact that some patients or their providers declined to participate or were reluctant to have penicillin allergy labels removed despite negative testing suggests opportunities for additional study to understand the barriers and facilitators of allergy delabeling. This descriptive study was limited by small sample size, but it provides a template for larger studies in the future. Additional randomized studies are needed to determine the impact of a PATS on antibiotic utilization, patient outcomes, and cost.

## Supplementary material

For supplementary material accompanying this paper visit http://doi.org/10.1017/ash.2023.144. click here to view supplementary material
