# Supplementary material for: Penicillin allergy evaluation in hospitalized patients with hematologic malignancy
Source: Antimicrob Steward Healthc Epidemiol. 2023 May 16;3(1):e92. doi: 10.1017/ash.2023.144 (PMC10204135; doi:10.1017/ash.2023.144)
Supplement: Supplementary file 1 [file ashsup.zip › S2732494X23001444sup001.pdf]

Inpatient Penicillin Skin Test Triage Questionnaire/Algorithm

Have you ever actually received a penicillin related antibiotic or amoxicillin and experienced a reaction?

NO

Why is penicillin allergy label on your chart? If unknown, see below**

1) Record Response
2) Confirm with on call provider
3) Remove allergy label if no history of PCN reaction

YES

What was the name of the penicillin-related antibiotic that caused your reaction?

Penicillin, Amoxicillin, or unknown

Other penicillin derivatives: amoxicillin-clavulanate (Augmentin), Ampicillin, nafcillin, oxacillin, dicloxacillin, piperacillin (Zosyn), ticarcillin

Update allergy tab with actual name of drug in chart

No skin test

When did the reaction occur? Record approximate Age.

≥ 10 years

What symptoms did you have with the reaction? **Circle/document all that apply**

< 10 years ago

Update chart & **Specify** the nature of the reaction.

No skin test

- Rash
- Hives
- Facial swelling
- Throat closing/swelling
- Low blood pressure
- Fainting (or light-headedness)
- Wheezing
- Shortness of breath

Unknown

Intolerance (e.g. nausea, vomiting, diarrhea or abdominal discomfort, headache, etc)

Symptoms consistent with Severe cutaneous drug reaction or meets other exclusion criteria***

Modify/clarify reaction in the chart.

No skin test

Have you taken penicillin or amoxicillin again since (after) you first had the reaction?

YES

Did you tolerate penicillin or amoxicillin?

NO

confirm not pregnant

Tolerated

Not tolerated, but reaction > 10 yrs ago

Perform skin test

1) Record Response (which drug, when administered)
2)Confirm with on call provider
3)Remove allergy label

Skin Prick Test

negative

Perform Intradermal Test

negative

Confirm with on-call provider and patient consents

positive

positive

Stop & Confirm with on-Call provider

Perform Oral Challenge

Inpatient Penicillin Skin Test Triage Questionnaire/Algorithm

**Why is penicillin allergy label on your chart? If unknown, ask the following

- Have you ever received penicillin or penicillin-related antibiotic without a reaction?
    - Yes- Confirm with provider and de-label
    - No/Unknown- Confirm patient is not pregnant, confirm with on call provider and skin test

*** **Symptoms consistent with Severe cutaneous drug reaction or meets other exclusion criteria**

- Signs and symptoms of Steven Johnson Syndrome (SJS), Toxic Epidermal Necrolysis (TEN), Drug Related Eosinophilia with Systemic Symptoms (DRESS)
- Heparin induced thrombocytopenia (HIT)
- Renal failure
- Liver failure
- Lung Injury
- Blistering type rashes
- Oral ulcerations
- Other severe reaction not consistent with IgE mediated reaction
