# Supplementary material for: Penicillin allergy evaluation in hospitalized patients with hematologic malignancy
Source: Antimicrob Steward Healthc Epidemiol. 2023 May 16;3(1):e92. doi: 10.1017/ash.2023.144 (PMC10204135; doi:10.1017/ash.2023.144)
Supplement: Supplementary file 1 [file ashsup.zip › S2732494X23001444sup002.pdf]

PCN Allergy Delabeling - Provider Survey

Please complete the survey below.

Thank you!

You are receiving this survey because you cared for patients on the hematology/oncology service during the conduct of our penicillin allergy delabeling initiative. This initiative used validated PCN allergy testing methods to remove inappropriate allergy labeling.

The goal of the below survey is to determine the feasibility and acceptability of this effort among providers (nurses, nurse practitioners, physicians, and pharmacists). We are interested in hearing your opinions about this project and suggestions for improvement. Your responses will remain anonymous and confidential. The survey will take approximately 10 minutes to complete. Thank you for taking the time to share your opinions.

What best describes your clinical role?

- ◯ Nurse
- ◯ Nurse practitioner or physician assistant
- ◯ Resident physician
- ◯ Attending physician
- ◯ Pharmacist
- ◯ Other

If you selected "other," please specify your role here

____

Did a patient in your care undergo penicillin allergy testing as part of this pilot study?

- ◯ Yes
- ◯ No

If you were approached regarding participation of one of your patients, and he/she did not end up participating, what was the reason (select all that apply)?

- ☐ Not applicable (I was not approached or was unaware of this study)-survey ends
- ☐ I did not have enough information about this project
- ☐ This intervention was not a priority
- ☐ I doubted whether the intervention would improve patient care
- ☐ I thought the intervention would take too much time
- ☐ I felt my patients were too sick
- ☐ My patient(s) were receiving other treatments or were involved in other trials which I felt conflicted with penicillin testing
- ☐ Other (type in response)
- ☐ Not applicable (all of my eligible patients participated)

Please explain "Other":

____

Approximately how many of your patients underwent penicillin testing / delabeling as part of this study?

- ◯ 1
- ◯ 2-5
- ◯ 6-10

How would you rate your understanding of the purpose of this study?	◯ Very good<br>◯ Good<br>◯ Poor<br>◯ Very poor
How would you rate the communication between the study team and the clinical care team as part of this study?	◯ Very clear<br>◯ Moderately clear<br>◯ Moderately unclear<br>◯ Very unclear
If applicable, please explain what parts of the process were unclear or confusing.	__________________________________
How receptive would you say your clinical team was to patients undergoing penicillin testing?	◯ Completely receptive<br>◯ Moderately receptive<br>◯ Moderately unreceptive<br>◯ Completely unreceptive
If applicable, please explain why your team was not completely receptive or comfortable with patients undergoing penicillin testing.	__________________________________
How receptive would you say your clinical team was to patients having penicillin allergy labels removed based on negative penicillin testing results?	◯ Completely receptive<br>◯ Moderately receptive<br>◯ Moderately unreceptive<br>◯ Completely unreceptive
If applicable, why was your clinical team not completely comfortable with patients having penicillin allergy labels removed despite negative testing?	__________________________________
How receptive would you say your patients were to having penicillin allergy labels removed based on negative penicillin testing results?	◯ Completely receptive<br>◯ Moderately receptive<br>◯ Moderately unreceptive<br>◯ Completely unreceptive
On occasions where patients refused or were reluctant to be delabeled despite negative testing, what were reasons they gave for not wanting to be delabeled? Please select all that apply:	☐ Patient did not understand the purpose of allergy delabeling<br>☐ Patient was afraid he or she might actually be allergic despite negative testing<br>☐ Patient did not understand why the allergy label would be removed<br>☐ Patient did not seem to trust the process used to determine absence of penicillin allergy<br>☐ Patient preferred to make the decision in the presence of his or her physician<br>☐ Patient would never want the allergy label removed no matter what<br>☐ Other reason(s) (please type in response)<br>☐ I don't recall
Please explain "Other":	__________________________________

How comfortable are you with using negative penicillin testing results to guide medical management in your patients? In other words, how comfortable would you be prescribing penicillin to a patient who had negative penicillin allergy testing (even if the patient previously had a labeled PCN allergy)?	◯ Completely comfortable<br>◯ Moderately comfortable<br>◯ Moderately uncomfortable<br>◯ Completely uncomfortable
If applicable, please explain why you are not completely comfortable prescribing penicillin to a patient despite negative penicillin allergy testing.	______________________________
How valuable is this intervention for your inpatients with hematologic malignancies?	◯ Very valuable<br>◯ Somewhat valuable<br>◯ Not valuable at all<br>◯ Harmful
Please explain why you think this intervention is valuable or not valuable.	______________________________
Would you like to see a team dedicated to performing penicillin testing on admitted patients routinely in the future?	◯ Yes, this would be very valuable<br>◯ Yes, this would be somewhat valuable<br>◯ No, this would not be valuable<br>◯ No, this would be harmful
If you have additional comments about this study or the idea of having a "penicillin allergy delabeling team" performing routine testing on inpatients, please provide those comments here. Thank you!	______________________________
